# Functional diversity of inhibitors tackling the differentiation blockage of MLL-rearranged leukemia

**DOI:** 10.1186/s13045-019-0749-y

**Published:** 2019-06-28

**Authors:** Krzysztof Brzezinka, Ekaterina Nevedomskaya, Ralf Lesche, Michael Steckel, Ashley L. Eheim, Andrea Haegebarth, Carlo Stresemann

**Affiliations:** Pharmaceuticals, Research & Development, Bayer AG, Muellerstrasse 178, 13353 Berlin, Germany

**Keywords:** Acute myeloid leukemia (AML), Acute lymphoblastic leukemia (ALL), MLL-fusion, Menin-MLL, DOT1L, DHODH, P-TEFb, BET, Small molecule inhibitors

## Abstract

**Introduction:**

The chromosomal rearrangements of the mixed-lineage leukemia gene MLL (KMT2A) have been extensively characterized as a potent oncogenic driver in leukemia. For its oncogenic function, most MLL-fusion proteins exploit the multienzyme super elongation complex leading to elevated expression of MLL target genes. High expression of MLL target genes overwrites the normal hematopoietic differentiation program, resulting in undifferentiated blasts characterized by the capacity to self-renew. Although extensive resources devoted to increased understanding of therapeutic targets to overcome de-differentiation in ALL/AML, the inter-dependencies of targets are still not well described. The majority of inhibitors potentially interfering with MLL-fusion protein driven transformation have been characterized in individual studies, which so far hindered their direct cross-comparison.

**Methods:**

In our study, we characterized head-to-head clinical stage inhibitors for BET, DHODH, DOT1L as well as two novel inhibitors for CDK9 and the Menin-MLL interaction with a focus on differentiation induction. We profiled those inhibitors for global gene expression effects in a large cell line panel and examined cellular responses such as inhibition of proliferation, apoptosis induction, cell cycle arrest, surface marker expression, morphological phenotype changes, and phagocytosis as functional differentiation readout. We also verified the combination potential of those inhibitors on proliferation and differentiation level.

**Results:**

Our analysis revealed significant differences in differentiation induction and in modulating MLL-fusion target gene expression. We observed Menin-MLL and DOT1L inhibitors act very specifically on MLL-fused leukemia cell lines, whereas inhibitors of BET, DHODH and P-TEFb have strong effects beyond MLL-fusions. Significant differentiation effects were detected for Menin-MLL, DOT1L, and DHODH inhibitors, whereas BET and CDK9 inhibitors primarily induced apoptosis in AML/ALL cancer models. For the first time, we explored combination potential of the abovementioned inhibitors with regards to overcoming the differentiation blockage.

**Conclusion:**

Our findings show substantial diversity in the molecular activities of those inhibitors and provide valuable insights into the further developmental potential as single agents or in combinations in MLL-fused leukemia.

**Electronic supplementary material:**

The online version of this article (10.1186/s13045-019-0749-y) contains supplementary material, which is available to authorized users.

## Background

Chromosomal rearrangements of the lysine methyltransferase 2A (KMT2A), known also as mixed-lineage leukemia (MLL) gene, are associated with high-risk infant, pediatric, adult, and therapy-induced acute leukemia. In infant and early childhood, acute leukemia is the most prevalent cancer and very often can be addressed with available therapeutics. A significant exception are patients genetically defined by MLL-fusions, where for most fusions, a worse prognosis [1] is underscoring the need for improved treatment options.

MLL associated genomic changes are balanced chromosomal translocations which result in an in-frame fusion of the MLL1 protein with a nuclear protein often involved in transcriptional elongation. So far, more than 130 different chromosomal rearrangements have been identified, but four of the most frequent fusion partners (AF4, AF9, ENL, and AF10) account for more than 70% of all observed rearrangements in patients [2]. While the diversity of observed fusions in patients suggests many disparate genetic subtypes, a common mode of action has been proposed for the oncogenic function of most frequently observed direct fusion (MLL-X) proteins [3]. These proteins essentially combine the target gene binding properties of the MLL1 protein with the capacity to trigger efficient transcriptional elongation by RNA polymerase II (RNAPII) recruitment. With the aforementioned properties, the MLL-fusion acts as the dominant transcriptional regulator which disrupts differentiation and promotes leukemogenesis [4, 5]. Wild-type MLL1 is responsible for the tissue-specific epigenetic regulation of homeotic gene expression in differentiation and development [6]. Catalytic SET domain is lost in the direct (MLL-X) fusion proteins, while the N-terminal DNA-binding domains and the capability to interact with recruiting co-factors, such as MENIN, are retained. The C-terminal part of different MLL1 fusion proteins is capable of recruiting a large multiprotein machinery (“super elongation complex” (SEC)) involved in activation of RNAPII for transcriptional elongation [7]. The mechanistic consequence of the SEC complex recruitment is an increased expression of MLL1 target genes leading to impaired differentiation. It has been shown that MLL-fusions exhibit their transforming capacity largely through upregulation of *HOX* genes [8, 9], especially HOXA9 and MEIS1 [10–12]. Normally, *HOXA9* and *MEIS1* are expressed at higher levels in stem cells and early lineage progenitors, and expression levels are downregulated with the process of differentiation [13]. Aberrant expression of *HOX* genes by the fusion induces a differentiation blockade resulting in leukemic cells with stem cell-like characteristics and increased self-renewal properties, growth, and survival advantages [14–16]. Since this differentiation blockade is an essential pathomechanism of MLL-fusion proteins, different therapeutic targets, whose inhibition might lead to terminal differentiation and reversal of the leukemia-initiating cells, have been suggested [1]. Notably, inhibitors that target core transcriptional proteins are of high interest, since they potentially interfere with the aberrant transcriptional elongation machinery and the leukemic gene expression program. Therefore, inhibitors against the kinase P-TEFb (CDK9/CyclinT1) [17], the histone methyltransferases DOT1L [18], and the bromodomain and extra-terminal domain (BET) family of proteins [19] are currently in clinical testing for AML. Another rather new strategy is the inhibition of the recruitment of the MLL-fusion and associated complex to the target genes. For this propose, inhibitors of the MENIN-MLL interaction have been described and are currently in pre-clinical evaluation [20–22]. Based on a phenotypic screening approach aimed towards HoxA9 regulation, inhibitors of the dihydroorotate dehydrogenase (DHODH) have emerged as an additional new strategy to overcome the differentiation blockade [23]. Despite initial positive pre-clinical evaluation of inhibitors against those targets in fused models of AML/ALL, first data on clinical activity of P-TEFb, BET, and DOT1L first-generation inhibitors are still awaiting true clinical proof of concept [19].

Here, we analyzed how inhibitors of some emerging therapeutic targets impact the differentiation blockade induced by the MLL-fusion in a comprehensive benchmark study. A better understanding of the differentiation effects could facilitate the further development and clinical translation of these novel agents. Therefore, in our study, we analyzed OTX015 (BET inhibitor) [24], Brequinar (DHODH inhibitor) [25], EPZ-5676 (DOT1L inhibitor) [26], and BAY 1251152 (novel first-in-class selective CDK9/P-TEFb inhibitor) [27], all representing clinical-stage small molecules (Table 1). Since MENIN-MLL inhibitors are not yet in clinical development, we additionally tested BAY-155, a novel potent and selective inhibitor derived from an in house program (further information see Additional file 1: Table S1) [28]. All different inhibitors were benchmarked for their capabilities to overcome the differentiation blockade, potential overlaps in transcriptional activities, selectivity for the MLL-fusion, and their combination potential.

**Table 1:**
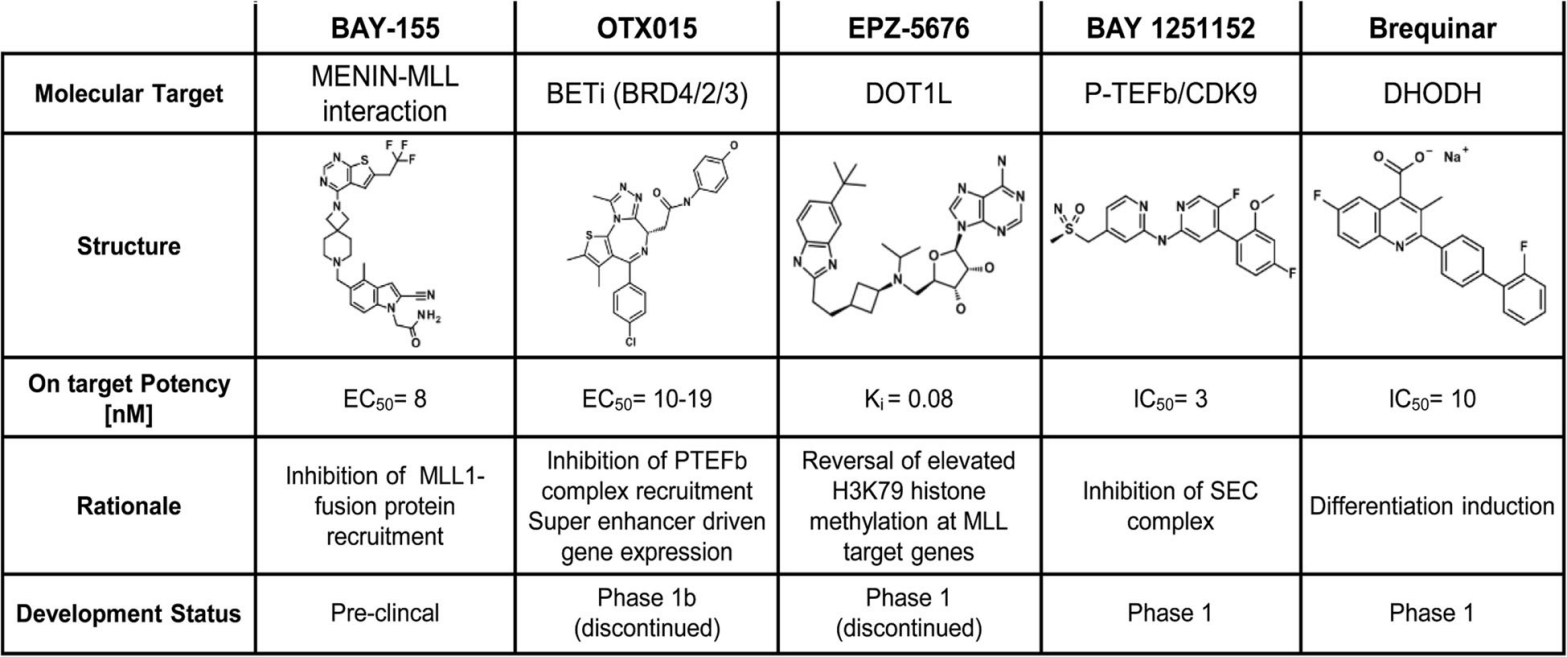
Inhibitors used in this study. Chemical structures of inhibitor used in this study tackling Menin-MLL1 interaction, BRD4/2/3, DOT1L, CDK9, and DHODH active sites, with respective biochemical IC_50_, rationale and current developmental status

## Materials and methods

### Cell lines

HL-60 cells were obtained from NCI 60-Panel. Jurkat and MV4-11 cells were obtained from ATCC. OCI-AML5, RS4;11, SEM, ML-2, MOLM-13, MOLM14, NOMO-1, OCI-AML2, KOPN-8, EOL-1, and OCI-AML3 cells were obtained from the Deutsche Sammlung von Mikroorganismen und Zellkulturen (DSMZ, Braunschweig, Germany). All used cells were cultured in the appropriate media and conditions.

### Inhibitors

All inhibitors used in this study were synthesized in-house (Bayer AG). BAY-155 was synthesized according to the methods outlined in patent application WO2017207387A1. Inhibitor concentrations for EPZ-5676, Brequinar, and OTX015 used in this in vitro study are lower as plasma concentrations measured in clinical studies [24, 26, 29]. Plasma concentrations of BAY 1251152 in humans are not yet reported.

### Cell proliferation

Cells were seeded in the optimal growth medium at 4000–5000 cells/well in a 96 MTP and cultured 18–24 h before inhibitor treatment. Upon treatment with the indicated inhibitor, cells were cultured for 24 h, 96 h, and 168 h and effects on proliferation were determined using alamarBlue Cell Viability Reagent (Thermo Fisher Scientific, Waltham, MA, USA).

### Flow cytometry

Four thousand cells per well were seeded 24 h before they were treated with the indicated inhibitor in a 96 MTP. After 4 or 7 days of treatment, cells were washed with PBS and stained with CD11b - APC (BioLegend, San Diego, California, USA) and DAPI (Thermo Fisher Scientific, Waltham, Massachusetts, USA) or AnnexinV – FITC (BioLegend, San Diego, California, USA) and PI solution (Sigma-Aldrich St. Louis, Missouri, USA) using the FACS Canto II (BD Biosciences, Heidelberg, Germany) and data was analyzed with FACSDiva software.

### Cell cycle analysis

Cells were washed with PBS and fixed overnight at − 20 °C with 70% ethanol. Fixed cells were stained with PI solution (Sigma-Aldrich St. Louis, MO, USA) solution containing RNase A (Qiagen, Hilden, Germany). Fluorescence was measured with FACS Canto II (BD Biosciences, Heidelberg, Germany) flow cytometer and data was analyzed with FACSDiva software.

### Wright-Giemsa staining

Approximately 10,000 of cytospin prepared cells were air dried, fixed in 100% methanol for 1 min, stained in 100% in Wright-Giemsa staining solution (Sigma-Aldrich St. Louis, Missouri, USA) for 90 s, washed two times in deionized water, and air dried.

### Phagocytosis assay

After 7 days of treatment with the indicated inhibitor, cells were washed once with PBS and quantified. Ten thousand viable cells were resuspended in fresh media along with fluorescein-labeled heat-killed *Escherichia coli* BioParticles (Molecular Probes, Eugene, OR, USA) (100,000 units), incubated at 37 °C for 30 min and stained with CD11b - APC (BioLegend, San Diego, CA, USA ) and DAPI. Phagocytosis capability was measured with FACS Canto II (BD Biosciences, Heidelberg, Germany). Immunofluorescence of cytospin preparations was measured on LSM700 microscope (ZEISS, Oberkochen, Germany) using CD11b (APC), DAPI, and *E.coli* particles (FITC).

### Gene expression

Total RNA was isolated using RNeasy-Plus Mini kit (Qiagen, Hilden, Germany). RNA (1 μg) was reverse transcribed using SuperScript III First-Strand Synthesis SuperMix (Life Technologies, Carlsbad, CA, USA) and obtained cDNA was used for qRT-PCR at the TaqMan 7900HT Fast Real-Time PCR System (Applied Biosystems, Foster City, CA, USA) utilizing TaqMan Fast Advanced Master Mix (Life Technologies). Commercial primers used in this study are listed in Additional file 2: Materials and methods. RNA-seq study: cells were treated for 8 h (P-TEFb—0.05 μM, OTX015—1 μM), 24 h (BAY-155—2 μM, Brequinar—2 μM, DMSO—0.1%) and 96 h (EPZ-5676—3 μM, DMSO—0.1%) prior to RNA extraction using RNeasy-Plus Mini kit (Qiagen). Obtained RNA was used for library preparation (Illumina, San Diego, CA, USA. TruSeq Stranded mRNA Kit) and obtained libraries were sequenced (Illumina, HiSeq2500 HTv4, SR, dual-indexing, 50 cycles).

### Data analysis and statistical methods

RNA-seq reads were aligned to hg38 using STAR aligner. Gene expression was quantified using RSEM. Samples with less than 10 million reads aligning to the genome were excluded; protein-coding genes with more than 10 reads in more than three samples were used for the analysis (total samples *N* = 305; genes *N* = 15,007). DESeq2 was used to find genes differentially expressed upon treatment by inhibitors in either each cell line or in the group of sensitive cell lines, while controlling for differences between the cell lines. GSEA analysis was run on the pre-ranked list based on logFC in expression for each compound. To remove cell line-specific differences in PCA, average expression in the DMSO sample was subtracted for each corresponding cell line. Top 1000 variable genes were selected based on median absolute deviation. Data is available at GEO (https://www.ncbi.nlm.nih.gov/geo/) under accession number GSE125437.

### Immunoblotting

Western blot analysis was performed on cell lysates from at least 100,000 cells. Forty micrograms of whole cell protein extract was separated on 4–20% Tris-Glycine gels, transferred to 0.2-μm nitrocellulose membranes, and probed with anti-HEXIM1 (Bethyl, Montgomery, TX, USA) and β-ACTIN (Cell Signaling, Beverly, MA, USA) antibodies.

## Results

### Cross-comparison of inhibitor-induced cell proliferation and differentiation effects

**Fig. 1:**
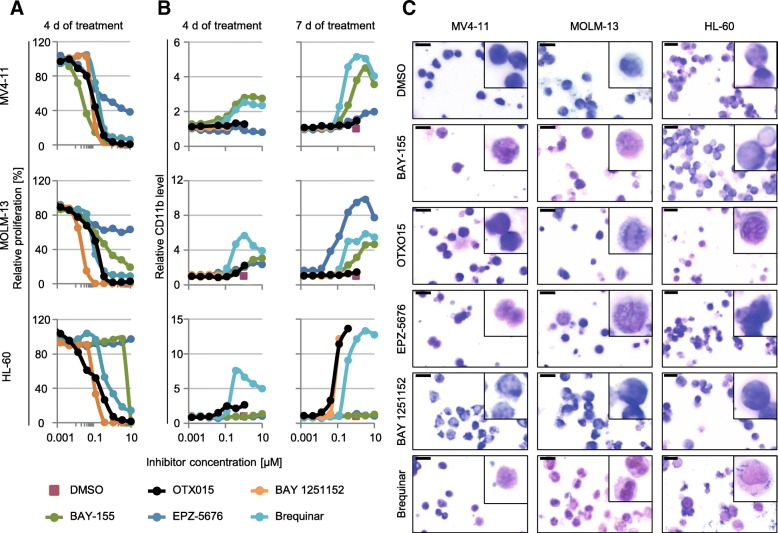
Comparing the inhibitors tackling the differentiation blockage in AML. **a** Proliferation inhibition effect of BAY-155, OTX015, EPZ-5676, BAY 1251152, and Brequinar in MV4-11, MOLM-13 and HL-60 cells after 4 days of treatment. Vehicle control (DMSO) of each cell line is set to 100%. Representative experiment of at least 3 biological replicates is shown. **b** Quantification of CD11b expression after BAY-155, OTX015, EPZ-5676, BAY 1251152, and Brequinar in MV4-11, MOLM-13, and HL-60 cell lines after 4 and 7 days of treatment detected with flow cytometry. Data represents median values from 10,000 living cells normalized to vehicle control (DMSO). **c** Wright-Giemsa-stained cytospins of MV4-11, MOLM-13, and HL-60 cells after 7 days of treatment with BAY-155 (0.05 μM, 0.5 μM, 2.5 μM, respectively), OTX015 (0.1 μM, 0.1 μM, 0.25 μM, respectively), EPZ-5676 (0.4 μM, 1 μM, 10 μM, respectively), BAY 1251152 (0.05 μM, 0.05 μM, 0.05 μM, respectively), and Brequinar (0.1 μM, 0.3 μM, 1 μM, respectively). In the top right corner of each image magnification of representative cells are shown. Black scale bar indicates 10 μm

As a first step to better understand the similarities and differences in the inhibition of selected MLL-fusion-associated therapeutic targets, we tested all selected inhibitors (Table 1) in cell proliferation assays in two MLL-fused (MV4-11, MOLM-13) and one non-fused AML (HL-60) cell line (Fig. 1a). We observed that OTX015, BAY 1251152, and Brequinar show strong anti-proliferation effects in all tested cell lines with IC_50_s between 30 nM and 140 nM. BAY-155 resulted in comparable strong effects in the MLL-fused cell lines. In contrast, the non-fused HL60 cell line was only affected with the 10 μM treatment. EPZ-5676 inhibited proliferation of the MLL-fused cell lines to 40–50% with no significant effects in HL-60. To further characterize the anti-proliferation effect, we assessed apoptosis induction (Additional file 1: Figure S1) and cell cycle effects (Additional file 1: Figure S2) using flow cytometry. For all tested inhibitors, a significant increase in apoptotic cells was detected at concentrations starting around their respective IC50 values confirming that apoptosis contributes to observed proliferation effects. Furthermore, in cell cycle analysis, BAY-155, OTX015, EPZ-5676, and BAY 1251152 treatment led to a decrease of cells in S and G2/M phase with increasing concentrations. In contrast, Brequinar treatment resulted in a slight S-phase arrest at lower concentrations (Additional file 1: Figure S2). Next, we investigated the ability to overcome the differentiation blockade of the AML cell lines. We performed flow cytometry analysis of CD11b protein expression as a surrogate maker for myeloid differentiation (Fig. 1b). BAY-155, Brequinar, EPZ-5676, or OTX015 treatments increased CD11b protein level in a dose and time-dependent manner in the MLL-fused cell lines. Interestingly, BAY-155 and EPZ-5676 did not increase CD11b level in the non-fused HL-60 cell line, whereas Brequinar, OTX015, and BAY 1251152 treatment did. However, BAY 1251152 only showed induction of CD11b within a limited concentration range close to IC_90_ after 7 days of treatment, corresponding to the very steep and concentration-dependent decrease in the proliferation rate. To examine differentiation on the morphologic level we performed Wright-Giemsa staining. We detected myeloid differentiation in a fraction of evaluated cells, which was reflected by typically associated morphology changes (decreased nuclei to cytoplasm ratio, indented/kidney shaped nuclei, and less-basophilic, vacuolated cytoplasm) (Fig. 1c). Morphological differentiation correlated with effects on CD11b induction with the exception of BAY 1251152 treatment, which did not show any significant effects on morphology. To further extend our study on morphological changes also to ALL models with or without MLL-fusion, we analyzed KOPN-8 (MLL-ENL) and Jurkat (MLL-WT) cells. Brequinar treatment also resulted in MLL-fusion independent induction of differentiation in ALL cell lines, whereas BAY-155 specifically affected differentiation of the MLL-ENL fused KOPN-8 model (Additional file 1: Figure S3). In summary, all tested inhibitors showed significant anti-proliferative effects on MLL-fused AML cell lines. However, only Brequinar, BAY-155, EPZ-5676, and partially OTX015 showed additional differentiation effects as denoted by CD11b induction and morphological changes. Furthermore, a functional impact of OTX015, Brequinar, and BAY 1251152 was also observed in HL-60 and Jurkat cells, suggesting that the molecular activities of those inhibitors are not restricted to the MLL-fusion pathway.

### Gene expression profiling in an AML/ALL cell line panel

**Fig. 2:**
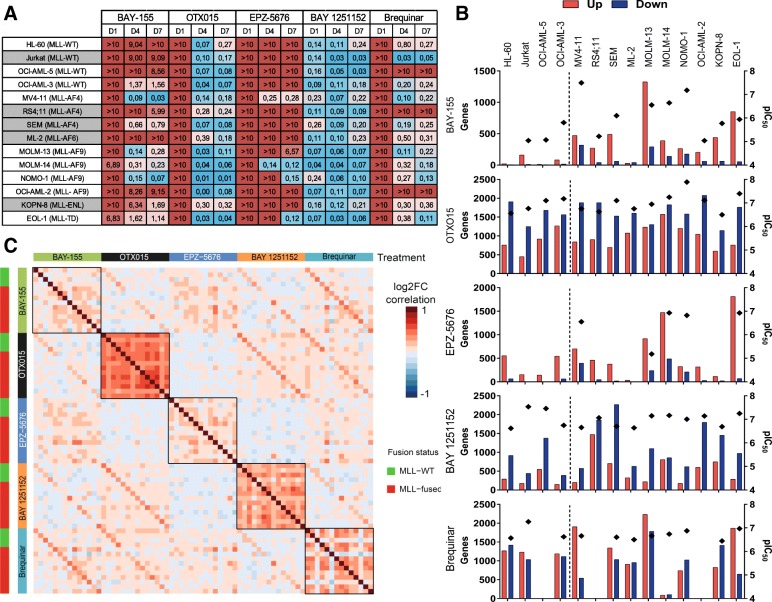
Inhibitor-induced differential gene expression and anti-proliferative effects in AML/ALL cells. **a** IC_50_ (μM) values of BAY-155, OTX015, EPZ-5676, BAY 1251152, and Brequinar after 1, 4, and 7 days of treatment. In the case where IC_50_ determination was beyond tested maximal concentration (10 μM), > 10 μM is used. ALL cell lines are indicated by gray name box shading. **b** Number of genes upregulated (red bars) and downregulated (blue bars) upon treatment by the indicated inhibitors in non-fused MLL-WT (left from the dashed line) and MLL-fused (right from the dashed line) models (log2FC > 1, FDR < 0.1). pIC_50_ (− log10 (IC_50_) in M) values (diamond shape) for the indicated inhibitors and cell models after 4 days of treatment. In the situation where pIC_50_ determination was beyond maximal concentration, no data point is shown. **c** Correlation of differential gene expression effects between inhibitors and cell models. Heatmap represents correlation of log2FC of gene expression grouped based on the inhibitor in all used cell lines ordered identically to **b**. Black boxes indicate cell line comparison for a single inhibitor

To further characterize the inhibitors, we performed a comprehensive gene expression analysis. We extended our cell line panel with additional 11 AML/ALL cell lines. To define appropriate treatment conditions for RNA sampling, we characterized all cell lines for proliferation effects induced by inhibitor treatment. Overall, as seen in the previous cellular experiments, BAY 1251152 and OTX015 followed by Brequinar had the strongest and most ubiquitous effects on proliferation, whereas BAY-155 and EPZ-5676 had significant (IC50 < 1 μM) proliferation effects specifically in selected MLL-fused models (Fig. 2a). Interestingly, treatment with BAY 1251152 could significantly inhibit cell proliferation of all tested cell lines already after 24 h of treatment, indicating an essential function of CDK9/PTEFb for cell viability. Based on these results, we defined the individual duration of inhibitor exposure and concentration to conditions without significant proliferation effects as we were especially interested in early and primary effects on gene expression. RNA-seq analysis showed all inhibitors affect expression of a high number of genes (log2FC > 1, FDR < 0.1), with the number depending on the cell line (Fig. 2b). In contradiction to the described functional roles of the MENIN-MLL interaction and DOT1L, BAY-155 and EPZ-5676 treatment resulted in a higher proportion of upregulated than downregulated genes. Moreover, both inhibitors had the strongest impact on gene expression in the MLL-fused models. In contrast, OTX015 and BAY 1251152 treatment led to a higher proportion of downregulated genes. Both inhibitors induced significant changes in all tested cell models irrespective of the MLL-fusion status. Treatment with Brequinar resulted in a more equal distribution of up- and downregulated genes in most cell line, while three cell lines did not respond on gene expression level, which corresponded to the matched proliferation results.

**Fig. 3:**
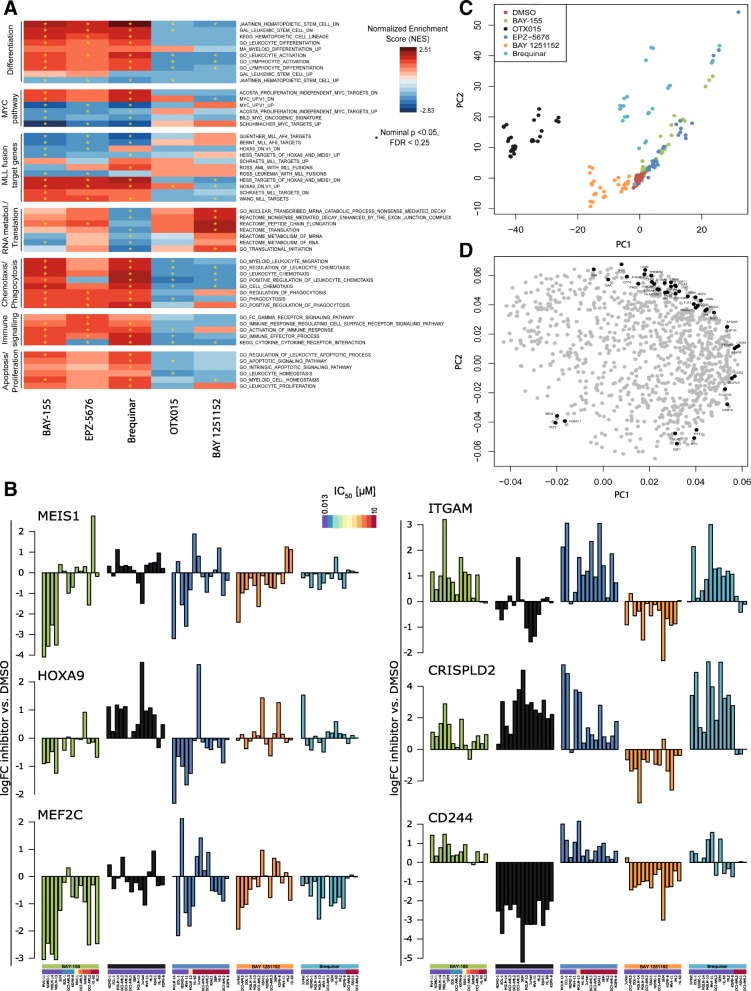
Gene set enrichment and principal component analyses. **a** Heatmap representing enrichment scores (NES) of different gene sets in GSEA. Yellow star indicates significant enrichment. **b** Analysis of logFC gene expression of indicated MLL target genes. Data is normalized to corresponding DMSO control and cell models are grouped based on their respective IC_50_ values. **c** Scores plot of the PCA based on the top thousand most variable genes in AML cell lines. Data adjusted to gene expression in vehicle (DMSO). **d** Loadings plot corresponding to the plot in **c**. Genes associated with AML differentiation are highlighted

Next, we analyzed the global gene expression effects in the context of (1) the individual inhibitor effect across different cell line models and (2) similarities of the inhibitors to each other (Fig. 2c). By analyzing the individual inhibitor effects across all models (Fig. 2c—black frames) OTX015, BAY 1251152, and Brequinar showed the most pronounced positive correlation across all responding cell line models (average coefficient of log2FC correlation 0.41, 0.26, and 0.3, respectively). This suggests a more universal mode of action independent of the MLL-fusion and underlying genetic background. Comparing the effects of different inhibitors, we found positive correlation between BAY-155–Brequinar and BAY 1251152–OTX015, which was most evident in the same cell line models (average coefficient of log2FC correlation 0.37 and 0.33). In a more detailed analysis of overlaps between only up- or downregulated genes, effects between BAY 1251152 and OTX015 were especially similar for gene downregulation indicating shared general activator functionality of P-TEFb and BRD4 (Additional file 1: Figure S4). As a next step, we evaluated which biological processes can be linked to the different gene expression responses. Therefore, we performed gene set enrichment analysis (GSEA) and principal component analysis (Fig. 3a and c, respectively) to address this question. The GSEA (Fig. 3a) shows that BAY-155, EPZ-5676, and Brequinar affect similar pathways in sensitive cell lines with a significant positive normalized enrichment score (NES) for myeloid and leukocyte differentiation induction. Moreover, those inhibitors significantly regulated gene sets involved in phagocytosis, chemotaxis, and immune response. In contrast, pathways regulated by MYC, MYB, MLL-fusion, HOXA9, or MEIS1 were negatively affected by all three inhibitors. Interestingly, BAY 1251152 and OTX015 negatively regulated gene sets associated with differentiation, phagocytosis, and immune signaling indicating a different mechanistic consequence for both inhibitors. On the other hand, treatment with BAY 1251152 positively regulated gene sets involved in the nonsense-mediated decay pathway and peptide chain elongation, while these gene sets were downregulated by Brequinar. Further, we analyzed several known MLL target genes which are found elevated or repressed in AML patients (Fig. 3b). We observed a strong correlation between BAY-155, EPZ-5676, and Brequinar in regulating MEF2C, ITGAM, CRISPLD2, and CD244. Interestingly, treatment with OTX015 and BAY 1251152 expression did not revert the MLL fusion-driven gene expression pattern. To better understand the similarities and differences between inhibitors effects, we used 1000 most variable genes in a principal component analysis (PCA) across all treated models. To eliminate cell line-specific differences, we centered all data on gene expression in the respective DMSO samples. Three distinct groups of samples can be seen in PC1-PC2 scores plot (Fig. 3c), where cells treated with BAY-155, EPZ-5676, and Brequinar cluster together and OTX015 as well BAY 1251152 separately. In the corresponding loadings plot, we could identify myeloid (Fig. 3d) and lymphoid (Additional file 1: Figure S5) cell surface markers as driving the difference between the samples. For the myeloid-derived cancer cell lines, we identified specific surface markers (e.g., ITGAM, ITGAX, CD68, CD86) usually present on monocytes, neutrophils, and macrophages, positively contributing to the BAY-155, EPZ-5676, and Brequinar group. For the lymphoid-derived cancer cell lines next to the specific surface markers (e.g., CD72, LAIR) associated with T/B-cells, we identified FLT3, HOXA9, MYC, and HEXIM1 as top genes driving the difference between the samples.

Interestingly, we observed HEXIM1 upregulation in all cell lines responding to Brequinar (Additional file 1: Figure S6a). In a previous study, HEXIM1 has been linked with nucleotide starvation, which was shown to sequester P-TEFb activity in melanoma [30]. Therefore, we hypothesized a direct relationship between DHODH inhibition and the elongation complex. As HEXIM1 function was associated with cell differentiation [31], we asked if HEXIM1 influences our inhibitor-induced AML differentiation. Upon HEXIM1 knockout, we observed a significant reduction of CD11b, MNDA and CD68 mRNA, and CD11b protein level after Brequinar treatment (Additional file 1: Figure S6b–d). Interestingly, induction of MNDA, LYZ, and CD68 gene expression after OTX015 treatment were also significantly reduced. This confirms the role of HEXIM1 in differentiation effects mediated by BET or DHODH inhibition. In summary, treatment with OTX015 and Brequinar showed the most pronounced and universal effects over all tested/responding cell lines. BAY-155 was in average more active in MLL-fused models. GSEA and PCA analysis of early global gene expression effects confirmed differentiation induced by treatment with of BAY-155, Brequinar, and EPZ-5676.

### Long-term treatment and phagocytosis assay as surrogate for functional differentiation

**Fig. 4:**
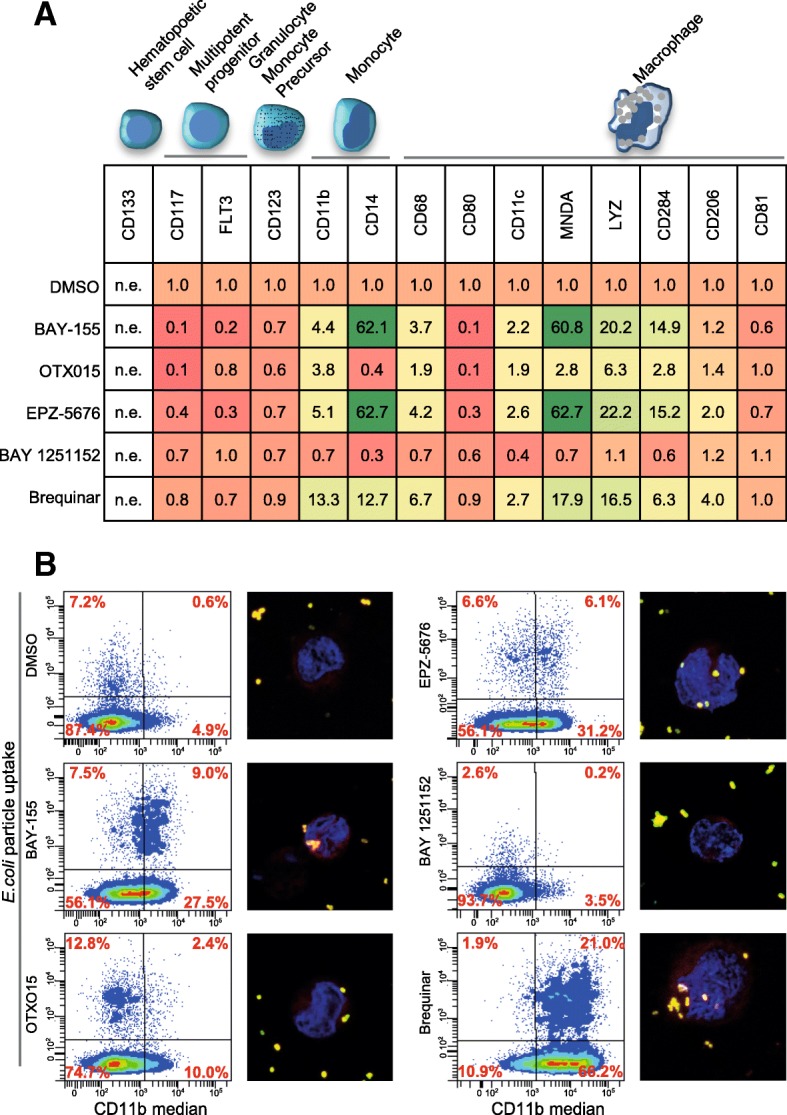
Phagocytosis of *E.coli* particles after inhibitor-induced differentiation. **a** qRT-PCR analysis of representative surface markers and genes associated with hematopoietic differentiation in MOLM-13 cells after 7 days of treatment with BAY-155 (3 μM), OTX015 (0.2 μM), EPZ-5676 (6 μM), BAY 1251152 (0.05 μM), and Brequinar (3 μM). Data presented is an average of three biological replicates normalized to vehicle control (DMSO). n.e. not expressed. **b** Flow cytometry scatter plots (1st and 3rd left column) showing the distribution of CD11b (APC) and *E.coli* (FITC labeled) staining of viable MOLM-13 cells after 7 days of treatment with BAY-155, (3 μM) OTX015 (0.2 μM), EPZ-5676 (6 μM), BAY 1251152 (0.05 μM), and Brequinar (3 μM). Representative experiment of three biological replicates is shown. Immunofluorescence staining (2nd and 4th left column) of MOLM-13 cells after 7 days of treatment with BAY-155, (3 μM) OTX015 (0.2 μM), EPZ-5676 (6 μM), BAY 1251152 (0.05 μM), and Brequinar (3 μM). Representative merge image of CD11b (red), *E.coli* particles (yellow), and nucleus (blue) is shown

Short-term treatment with BAY-155, EPZ-5676, and Brequinar was sufficient to induce expression of genes associated with differentiation. This led us to hypothesize that long-term treatment could differentiate to a more terminal stage thereby recovering normal cell function. Thus, we analyzed a number of cell surface markers and other genes linked with myeloid differentiation on the gene expression level after a prolonged exposure of 7 days of treatment (Fig. 4a). We observed that all tested inhibitors decreased the expression of markers associated with multipotent progenitors and Granulocyte Monocyte precursors (CD117, FLT3, and CD123), with BAY-155 and EPZ-5676 treatment having the strongest effect. Furthermore, both inhibitors showed upregulation of Monocyte CD11b and CD14 markers and moderate to strong upregulation of macrophage-associated marker genes. Similar effects on the differentiation marker genes were detected after Brequinar treatment. Surprisingly, also OTX015 showed after prolonged exposure significant, albeit weaker, induction of those marker genes.

In contrast to that, BAY-155 and EPZ-5676 treatment in HL60 (MLL-WT) (Additional file 1: Figure S7) did not modulate differentiation-associated marker genes. In HL60, Brequinar and OTX015 showed significant upregulation of some markers (e.g., CD11b, LYZ). BAY 1251152 treatment resulted in downregulation of majority of tested genes in MOLM-13 and HL60. Next, we were interested if the observed differentiation effects would translate into regaining functional properties of myeloid differentiated cells. For this purpose, we tested the capabilities of MOLM-13 cells to phagocyte *E.coli* particles. As shown in Fig. 4b, Brequinar treatment increased CD11b and phagocytosis level most effectively with 30% of CD11b positive cells showing particle uptake. Increased phagocytosis activity combined with CD11b induction were observed to a lesser extent for BAY-155 and EPZ-5676. OTX015 induced CD11b and phagocytosis activity only slightly. Altogether, we observed that prolonged treatment with Brequinar, BAY-155, and EPZ-5676 induces a number of differentiation-associated markers and a partial regain of cellular functionality in vitro.

### Combination potential of different inhibitors

**Fig. 5:**
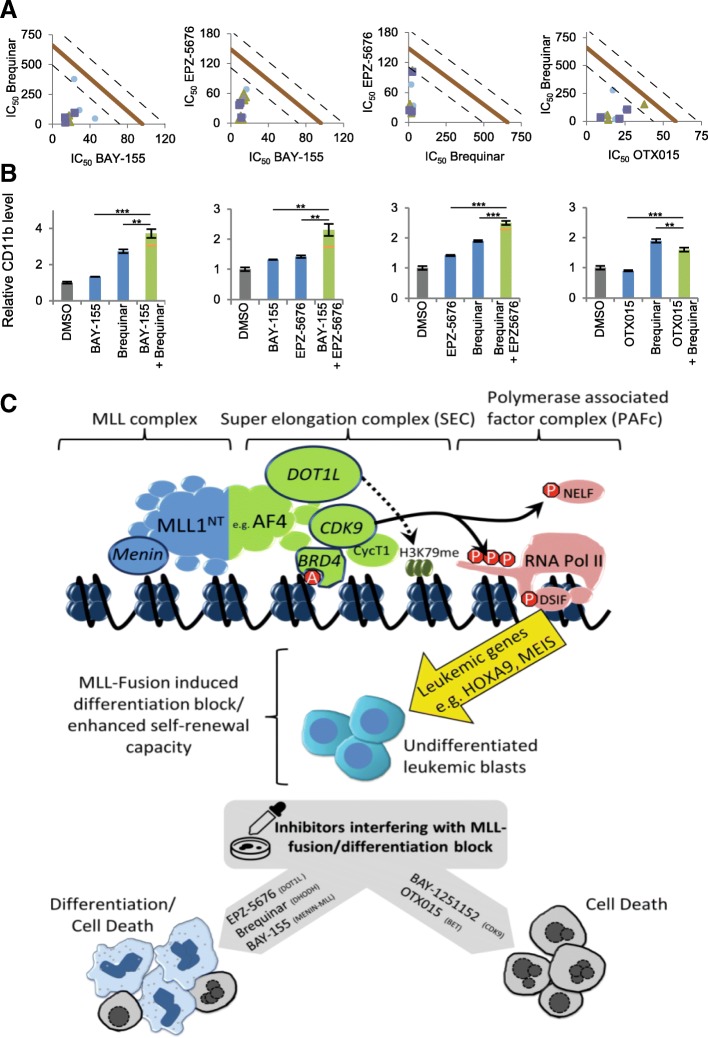
Analysis of combinations effects on proliferation and CD11b expression levels. **a** IC_50_-based isobologram analysis of inhibitor combinations in MOLM-13 cells after 4 days of treatment. The diagonal lines indicate additivity. Experimental data points, represented by dots (square, triangle, and circle) indicate biological replicates. **b** CD11b expression level after BAY-155 (0.15 μM)–Brequinar (0.64 μM), BAY-155 (0.15 μM)–EPZ-5676 (0.64 μM), Brequinar (0.15 μM)–EPZ-5676 (0.64 μM), and Brequinar (0.15 μM)–OTX015 (0.15 μM) combinations in MOLM-13 cells after 4 days of treatment. Data shows a representative concentration. Bar charts show an average of three biological replicates, the orange line indicates predicted additive effect, error shows SD, statistics ***P < 0.001, **P < 0.01, *P < 0.05, n.s. *P* < 0.05, two-sided *t* test. **c** Graphical summary

Since all inhibitors used in this study potentially interfere at different stages with MLL-fusion proteins, they could potentially be combined to achieve superior effects. Therefore, we tested all possible combinations (10 combinations per cell line model) on cell proliferation and differentiation (Fig. 5 and Additional file 1: Figure S8 and S9) by using inhibitor-inhibitor concentration matrixes combined with IC_50_ evaluation. We observed clear anti-proliferative synergism for BAY-155 in combination with Brequinar (combination index, 0.27–0.64) and EPZ-5676 (combination index, 0.21–0.51) as well for Brequinar combined with EPZ-5676 (combination index, 0.32–0.97) (Fig. 5a). All three combinations resulted in significant differentiation synergisms (Fig. 5b). Interestingly, Brequinar used in combination with OTX015 showed clear anti-proliferative synergism (combination index, 0.28–0.71) with antagonistic differentiation effects (Fig. 5a, b). All other tested combinations resulted in anti-proliferative synergism or additivity but no differentiation synergisms effects (Additional file 1: Figures S8 and S9). In summary, we found synergistic effects on the differentiation level when BAY-155, Brequinar, and EPZ-5676 were combined.

## Discussion

The concept of differentiation therapy emerged in the late 1970s when retinoic acid (RA) cAMP, sodium butyrate, arsenic trioxide, and cytokines were proposed to treat acute promyelocytic leukemia (APL). Since then, several clinical studies have shown treatment benefits by using all-trans RA in combination with arsenic trioxide resulting in > 90% complete remission [32]. Nevertheless, effects are restricted to a specific chromosomal translocation *t* [15, 17] driving APL comprising 10% of all AML patients [16]. Therefore, new strategies tackling the differentiation blockade and self-renewal capacity of AML/ALL cells with different genetic alterations were proposed and are currently under clinical evaluation [33, 34].

In our comprehensive study in MLL-fused AML/ALL models, we have used inhibitors against CDK9 (BAY 1251152), DOT1L (EPZ-5676), BRD2/3/4 (OTX015), MENIN-MLL interaction (BAY-155), and DHODH (Brequinar). All these proteins have been associated with differentiation in AML/ALL [23, 31, 35–40], but since inhibitors for those protein targets have all been used so far under isolated experimental conditions, a direct comparison of their differentiation capacity was not possible. Therefore, we profiled those inhibitors head-to-head for gene expression effects in a large cell line panel. We further examined cellular responses such as inhibition of proliferation, apoptosis induction, cell cycle arrest, and phagocytosis as functional differentiation readout. Based on our results, we found clear differences in the differentiation capacity and specificity for MLL-fused AML/ALL cell lines of examined inhibitors (Fig. 5c).

We observed that BAY-155 and EPZ-5676 treatment led to anti-proliferative effects, transcriptional changes, and differentiation exclusively in the MLL-fused AML models. This data confirms a driver function of Menin and DOT1L especially in the MLL-fusion-induced de-differentiation and increased self-renewal activity via aberrant transcriptional activation of master regulators (e.g., HOXA9, MEIS1, and MYB). Inhibiting expression of those stemness-associated master regulators by inhibition of Menin or DOT1L triggers expression of differentiation-associated genes. This could explain our observation of a higher number of upregulated genes after inhibitor treatment in contrast to the described activating function of those proteins. Menin is required for the recruitment of the MLL-fused protein, which co-recruits the elongation complex (AF4, P-TEFb, ENL, DOT1L, and BRD4) causing extension of H3K4me3 and H3K79me3 marks on transcribed gene bodies. DOT1L is the essential H3K79 methyltransferase, which creates extended H3K79 methylation and overwrites normal epigenetic regulation pattern [41]. As consequence, productive elongation of MLL-fusion target genes by RNAPII is promoted resulting in transcriptional reprograming and loss of cellular identity [42]. In a clinical phase I study, EPZ-5676 was evaluated in AML patients and a significant reduction of H3K79me2 on *HOXA9* and *MEIS1* was observed [26]. This observation also correlates with our gene expression analysis and previous reports. Interestingly, while comparing the effects of BAY-155 and EPZ-5676, it appears that blocking the recruitment of MLL-fusion complex is a more efficient way to induce transcriptional changes, differentiation, and cell killing than inhibiting DOT1L. Tackling the Menin-MLL interaction in MLL-fused AML/ALL induces overall very similar transcriptional changes as with inhibition of the DOT1L methyltransferase activity. Nevertheless, Menin-MLL inhibition resulted in significantly faster anti-proliferation and differentiation effects. Faster effects after the inhibition of the Menin-MLL interaction can be partially explained by the kinetics of the MLL-fusion as an oncogenic driver. The Menin-MLL interaction is mechanistically further upstream than the methylation activity of DOT1L [43]. Therefore, Menin-MLL inhibition leads to an overall reduced recruitment of ENL and other elongation factors (like DOT1L), which then leads to the observed suppression of HOXA10, MEIS1, and MYB, and upregulation of CD11b [44]. For DOT1L, it has been reported that both genetic and pharmacological targeting results in delayed (4–10 day) effects on transcriptional regulation and cell viability in AML [41, 45], which can be explained by the slow turnover rate of pre-existing H3K79 methylation [46]. Interestingly, we could detect proliferation and differentiation synergisms of BAY-155 and EPZ-5676 combination. This might be explained by the possibility that inhibition of Menin-MLL or DOT1L alone does not fully inhibit all MLL-fusion activities. Possibly, Menin independent recruitment or other SEC member (e.g., ENL) activities might promote transcriptional elongation independently from H3K79me [17]. Pharmacological inhibition of the Menin-MLL interaction appears to be selective to the MLL-fused AML/ALL with differentiation induction and anti-proliferation potential; however, this treatment option still awaits clinical evaluation.

Another approach in AML therapy conceived in the past years is blocking of multiple transformation pathways which are dependent on the P-TEFb function via BET and CDK9 inhibition. Both targets were shown to be critical for AML/ALL cell viability mainly through regulating MYC, MYB, and MCL1 levels [17, 37, 47]. While genetic and pharmacological BRD4 inhibition was linked to cell differentiation [47], a direct inhibition of CDK9 activity results in differential responses. Our study results confirm strong cell killing activity of both inhibitors and transcriptional inhibition of CDK9/BET regulated target genes [17, 48]. In our study, only BET but not CDK9 inhibition resulted in cell differentiation on transcriptional and morphological level. However, early transcriptional profiling of OTX015 did not show any significant positive effects on AML/ALL differentiation associated pathways. When applied for several days at higher concentrations OTX015 induces differentiation effects independent from the MLL-fusion, which hints to differentiation as secondary to primary gene expression effects. One explanation for the delayed effect of OTX015 on differentiation might be the direct downregulation of transcription factors MYB and MYC. It has been reported that their ectopic expression is inhibiting differentiation in a number of cell lines and primary cells [49, 50]. Additionally, OTX015 modulates the largest number of genes, even at the very early time point tested, of all inhibitors, which indicates a substantial effect on the global gene expression network. Those expression changes resulted in differentiation effects only in a limited number of cells but overall resulted in very robust anti-proliferation effects. Strong global effects on transcription might also be the reason for the inability of CDK9 inhibition to induce differentiation. Inhibition of proliferation and apoptosis induction is the dominant effect of CDK9 inhibitor, and cells are killed before a potential interference with the MLL-fusion leads to differentiation. Currently, BAY 1251152 undergoes phase I clinical evaluation with no final report yet. Initial pharmacodynamics data analysis shows dose-dependent reduction of *MYC*, *PCNA*, and *MCL-1* levels, all being relevant for cancer cell survival [51]. Interestingly, OTX015 clinical trial performed in AML patients harboring a number of diverse driving mutations resulted in partial blast clearance and recovery of platelets. However, severe thrombocytopenia as dose-limiting effect was observed in patients with incomplete bone marrow failure [24]. Altogether, our cellular analysis for OTX015 and BAY 1251152 support the clinical observations and suggest that interfering with P-TEFb function via BET and CDK9 inhibition leads primary to strong anti-proliferation and apoptosis induction effects which are MLL-fusion independent.

Lastly, DHODH, an enzyme in the de novo synthesis of nucleotides, was shown to be critical for the self-renewal and proliferation capacity in a wide variety of AML models [23, 52]. Our data is significantly extending those findings by connecting the described differentiation phenotypes of Brequinar with global gene expression profiling and functional AML differentiation. Interestingly, tackling de novo pyrimidine biosynthesis leads to a pronounced effect on global gene expression but also to a very specific response in AML/ALL relevant pathways which is not restricted to MLL-fused models. Moreover, DHODH inhibition by Brequinar undergoes a phase I clinical reevaluation in AML patients after encouraging pre-clinical observations suggesting its role in differentiation [23, 52]. Furthermore, we have observed that Brequinar effect on gene expression is similar to the effects of BAY-155 and EPZ-5676 in MLL-fused models inducing more terminal differentiation. Brequinar in combination with BAY-155 or EPZ-5676 leads also to significant anti-proliferation and differentiation synergism, whereas combining Brequinar with OTX015 and BAY 1251152 induces exclusively anti-proliferation synergy. While nucleotide shortage induces stress and therefore explains proliferation inhibition and cell cycle arrest, it is also reported to drive HEXIM1 expression [30]. Our data provides for the first time a direct link between HEXIM1- and Brequinar-induced nucleotide stress leading to AML/ALL differentiation. In summary, our novel findings extend the understanding of Brequinar-mediated AML/ALL differentiation and explore some of possible combinations. Altogether, based on our results, inhibiting Menin-MLL together with DOT1L might allow for a more efficient and MLL-fusion-specific induction of differentiation and apoptosis. In contrast, BAY 1251152, OTX015, and Brequinar are significantly affecting also differentiation independent pathways (e.g., RNA metabolism/translation). This might limit their combination potential since expected treatment tolerability could be lowered.

In conclusion, these new findings enhance our understanding on the activity of used inhibitors of those emerging therapeutic targets in MLL-fusion-driven leukemia. Our novel findings give some valuable insights into their differentiation induction potential, which is a possible underestimated contribution of their therapeutic activities in AML/ALL.

## Additional files

Additional file 1: **Table S1.** Characterization of BAY-155 inhibitor. **Figure S1.** Apoptosis and cell death induced by BAY-155, OTX015, EPZ-5676, BAY 1251152 and Brequinar treatment. **Figure S2.** Cell cycle arrest induced by BAY-155, OTX015, EPZ-5676, BAY 1251152 and Brequinar treatment. **Figure S3.** Morphological differentiation induced by inhibitor treatment in ALL. **Figure S4.** Overlaps of up- and downregulated genes between different inhibitors and cell models. **Figure S5.** Principal component analysis of gene expression in ALL cell lines. Figure S6. HEXIM1 links DHODH inhibition with cell differentiation. **Figure S7.** Surface marker analysis after inhibitor induced differentiation in HL-60 cells. **Figure S8.** Analysis of combination effects of used inhibitors in MOLM-13 cells. **Figure S9.** Analysis of combination effects on proliferation of MV4-11 and HL-60 cells. (DOCX 13318 kb)

Additional file 2: TaqMan probes used in this study. (DOCX 13 kb)

## Data Availability

The data generated or analyzed during this study are included in the published article and its supplementary files. Gene expression data is available at GEO (https://www.ncbi.nlm.nih.gov/geo/) under accession number GSE125437.

## Abbreviations

ALL: Acute lymphoblastic leukemia
AML: Acute myeloid leukemia
APL: Acute promyelocytic leukemia
BET: Bromodomain and extraterminal domain
CDK9: Cyclin-dependent kinase 9
DHODH: Dihydroorotate dehydrogenase
DOT1L: DOT1-like histone H3K79 methyltransferase
GSEA: Gene set enrichment analysis
MLL: Mixed-lineage leukemia
NES: Normalized enrichment score
PCA: Principal component analysis
P-TEFb: Positive transcription elongation factor b
RA: Retinoid acid
RNAPII: RNA polymerase II
SEC: Super elongation complex
