# Supplementary material for: Functional diversity of inhibitors tackling the differentiation blockage of MLL-rearranged leukemia
Source: J Hematol Oncol. 2019 Jun 28;12:66. doi: 10.1186/s13045-019-0749-y (PMC6599250; doi:10.1186/s13045-019-0749-y)
Supplement: Supplementary file 1 — Table S1. Characterization of BAY-155 inhibitor. Figure S1. Apoptosis and cell death induced by BAY-155, OTX015, EPZ-5676, BAY 1251152 and Brequinar treatment. Figure S2. Cell cycle arrest induced by BAY-155, OTX015, EPZ-5676, BAY 1251152 and Brequinar treatment. Figure S3. Morphological differentiation induced by inhibitor treatment in ALL. Figure S4. Overlaps of up- and downregulated genes between different inhibitors and cell models. Figure S5. Principal component analysis of gene expression in ALL cell lines. Figure S6. HEXIM1 links DHODH inhibition with cell differentiation. Figure S7. Surface marker analysis after inhibitor induced differentiation in HL-60 cells. Figure S8. Analysis of combination effects of used inhibitors in MOLM-13 cells. Figure S9. Analysis of combination effects on proliferation of MV4-11 and HL-60 cells. (DOCX 13318 kb) [file 13045_2019_749_MOESM1_ESM.docx]

Supporting Information Data

Functional diversity of inhibitors tackling the differentiation blockage of MLL-rearranged leukemia

*Krzysztof Brzezinka^1^,* *Ekaterina Nevedomskaya^1^, Ralf Lesche^1^, Michael Steckel^1^, Ashley L. Eheim^1^, Andrea Haegebarth^1^, and Carlo Stresemann^*1^*

*^1^Bayer AG, Research & Development, Pharmaceuticals, Muellerstrasse 178, D-13353 Berlin, Germany*

^*^Corresponding author:carlo.stresemann@bayer.com**,** Tel: +49 30 468-12866

Supplementary Table

**Table S1.**

**Characterization of BAY-155 inhibitor.**

**
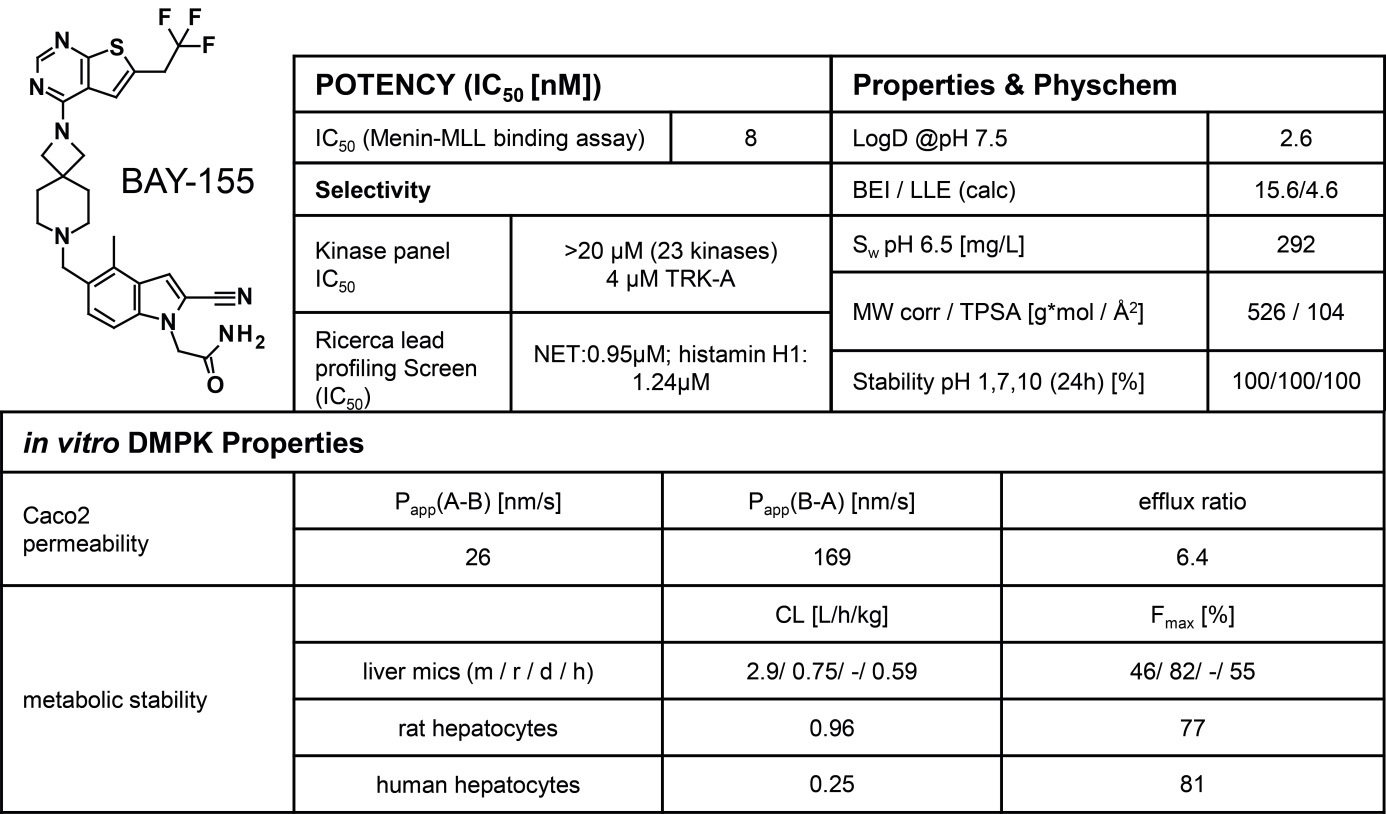
**

Supplementary Figures

**
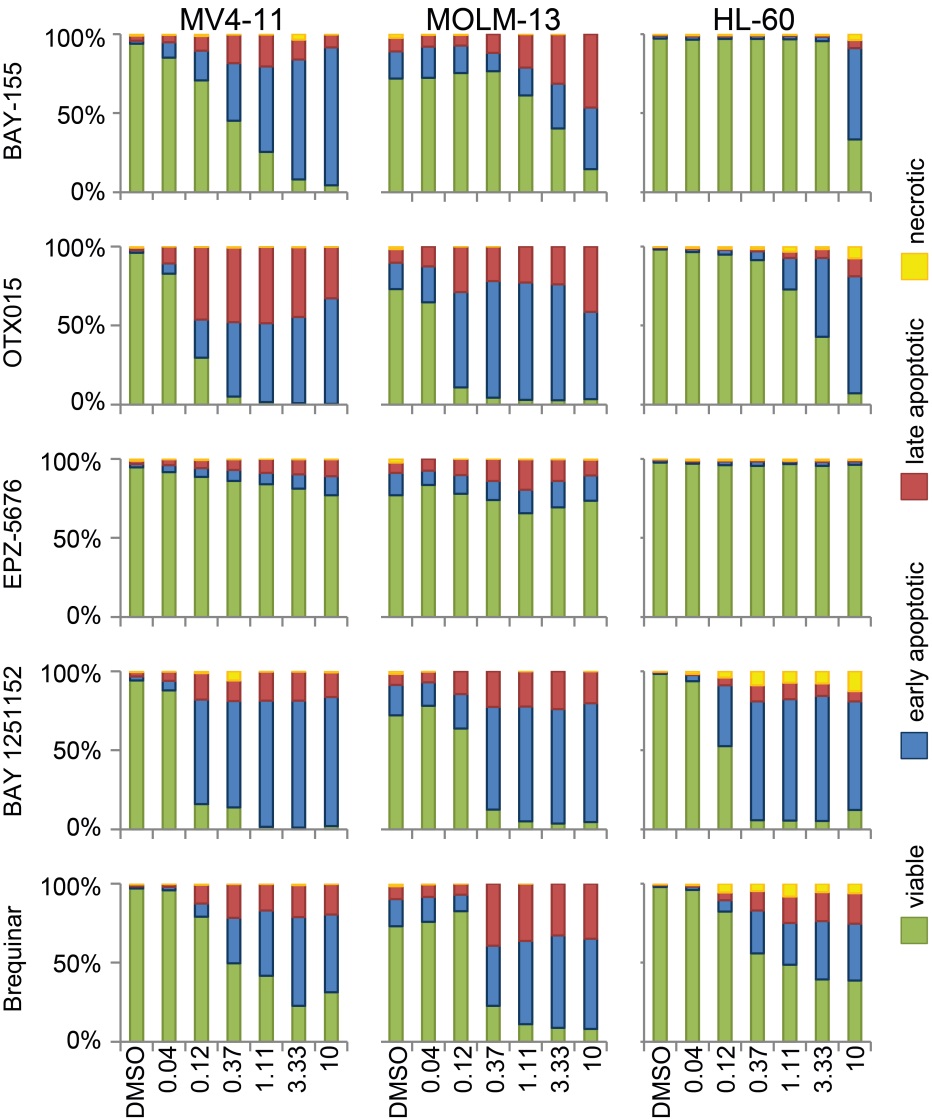
**

**Figure S1.**

**Apoptosis and cell death induced by BAY-155, OTX015, EPZ-5676, BAY 1251152 and Brequinar treatment.** Cell viability, necrosis, early and late apoptosis induced by BAY-155, OTX015, EPZ-5676, BAY 1251152, and Brequinar after 4 days of treatment in MV4-11, MOLM-13 and HL-60 cell lines. Quantification of Annexin V and Propidium Iodide (PI) staining was detected by flow cytometry. Data represents % of each cell state in relation to all single intact cell population.

**
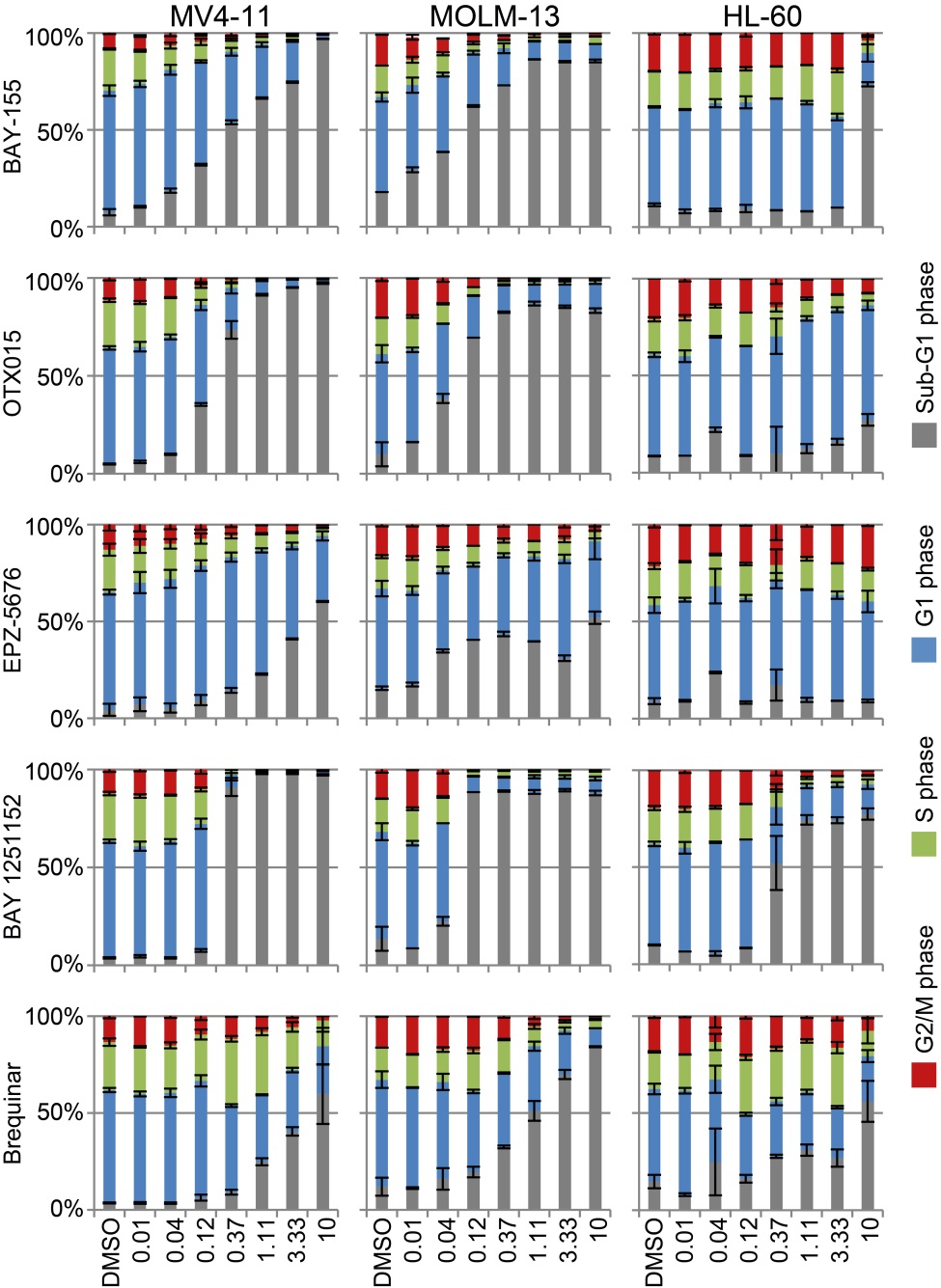
**

**Figure S2.**

**Cell cycle arrest induced by BAY-155, OTX015, EPZ-5676, BAY 1251152 and Brequinar treatment.** Cell-cycle analysis by quantification of propidium iodide DNA content after 4 days of treatment with BAY-155, OTX015, EPZ-5676, BAY 1251152, and Brequinar in MV4-11, MOLM-13 and HL-60 cell lines. Bar charts show an average of two biological replicates, error shows SD.


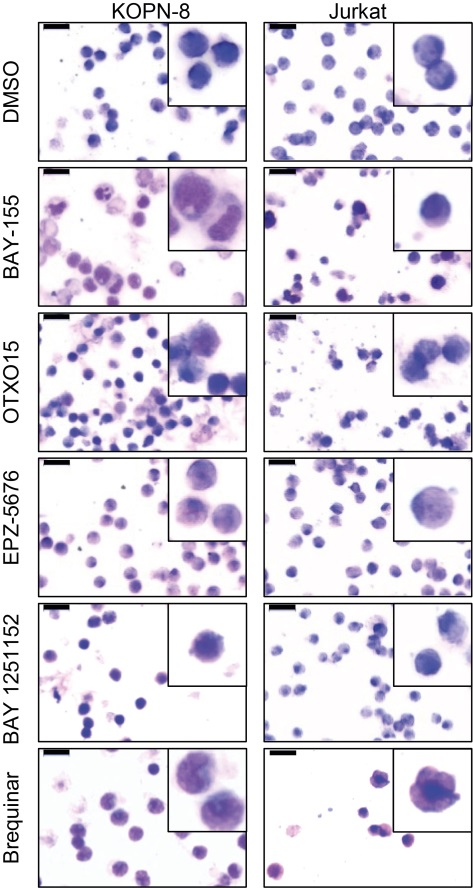


**Figure S3.**

**Morphological differentiation induced by inhibitor treatment in ALL.** Wright-Giemsa-stained cytospins of KOPN-8 and Jurkat cells after 7 days of treatment with BAY-155 (2.5 µM, 10 µM respectively), OTX015 (0.05 µM, 0.6 µM respectively), EPZ-5676 (10 µM, 10 µM respectively), BAY 1251152 (0.1 µM, 0.1 µM respectively) and Brequinar (1 µM, 0.2 µM respectively). In the top right corner of each image magnification of representative cells are shown. Black scale bar indicates 10 µm.


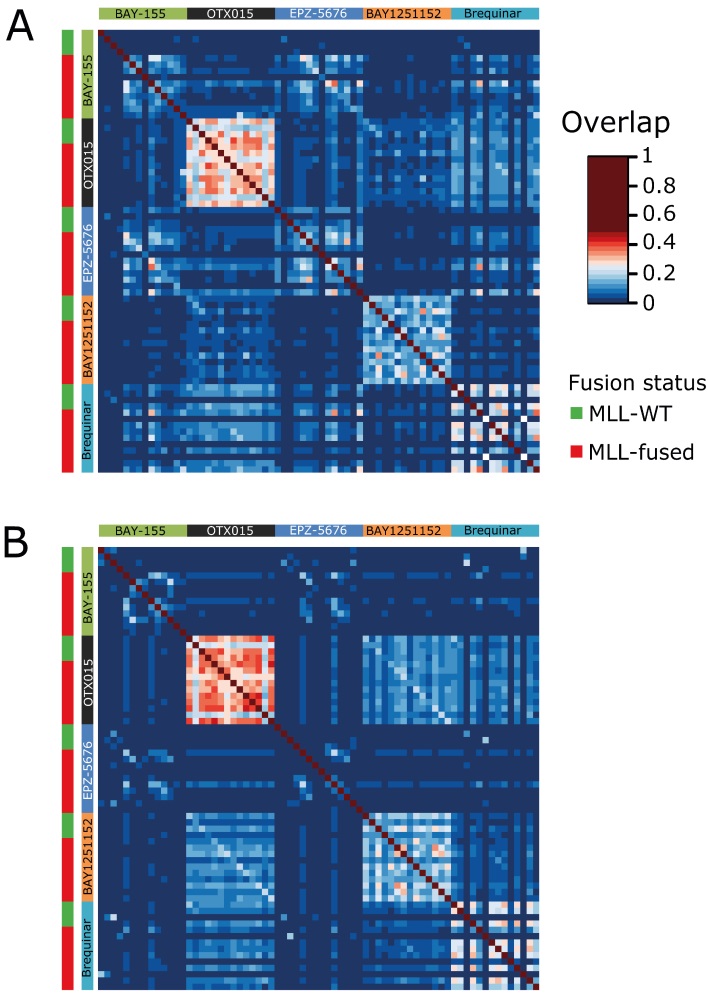


**Figure S4.**

**Overlaps of up- and down-regulated genes between different inhibitors and cell models.** Heatmap representing overlaps of up-regulated (A) and down-regulated (B) genes sorted by inhibitor. Overlap proportions are calculated as follows: Overlap = A∩B/(A_unique_ + A∩B + B_unique_), where A and B represent the conditions compared.


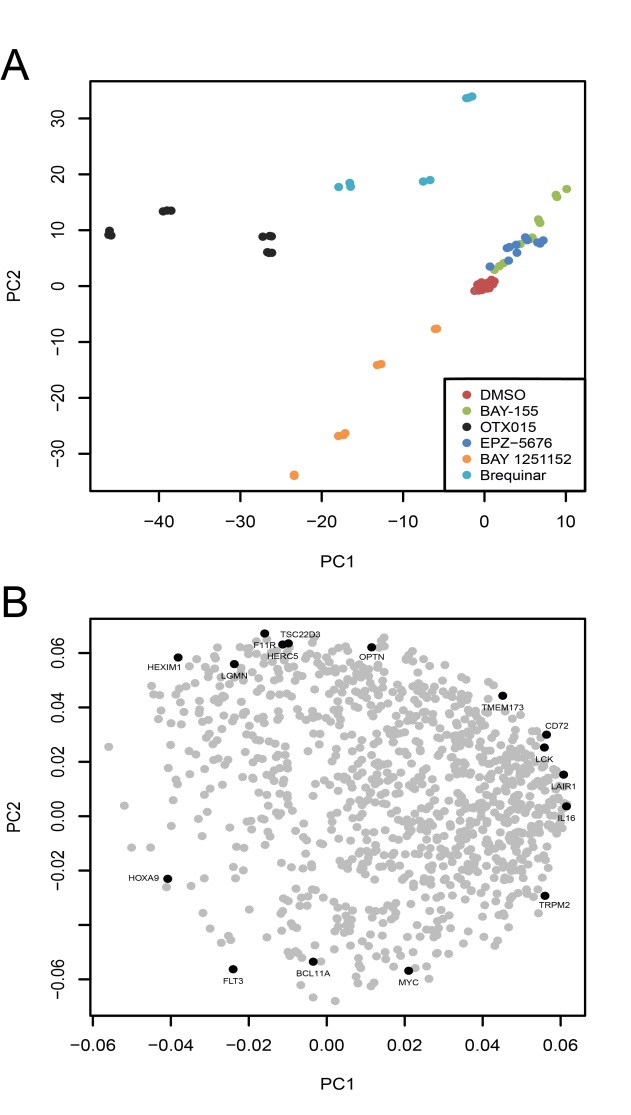


**Figure S5.**

**Principal component analysis of gene expression in** **ALL cell lines.** (A) Scores plot of the PCA based on the top thousand most variable genes in ALL cell lines. Data adjusted to gene expression in vehicle (DMSO). (C) Loadings plot corresponding to the plot in (B). Genes associated with ALL differentiation are highlighted.


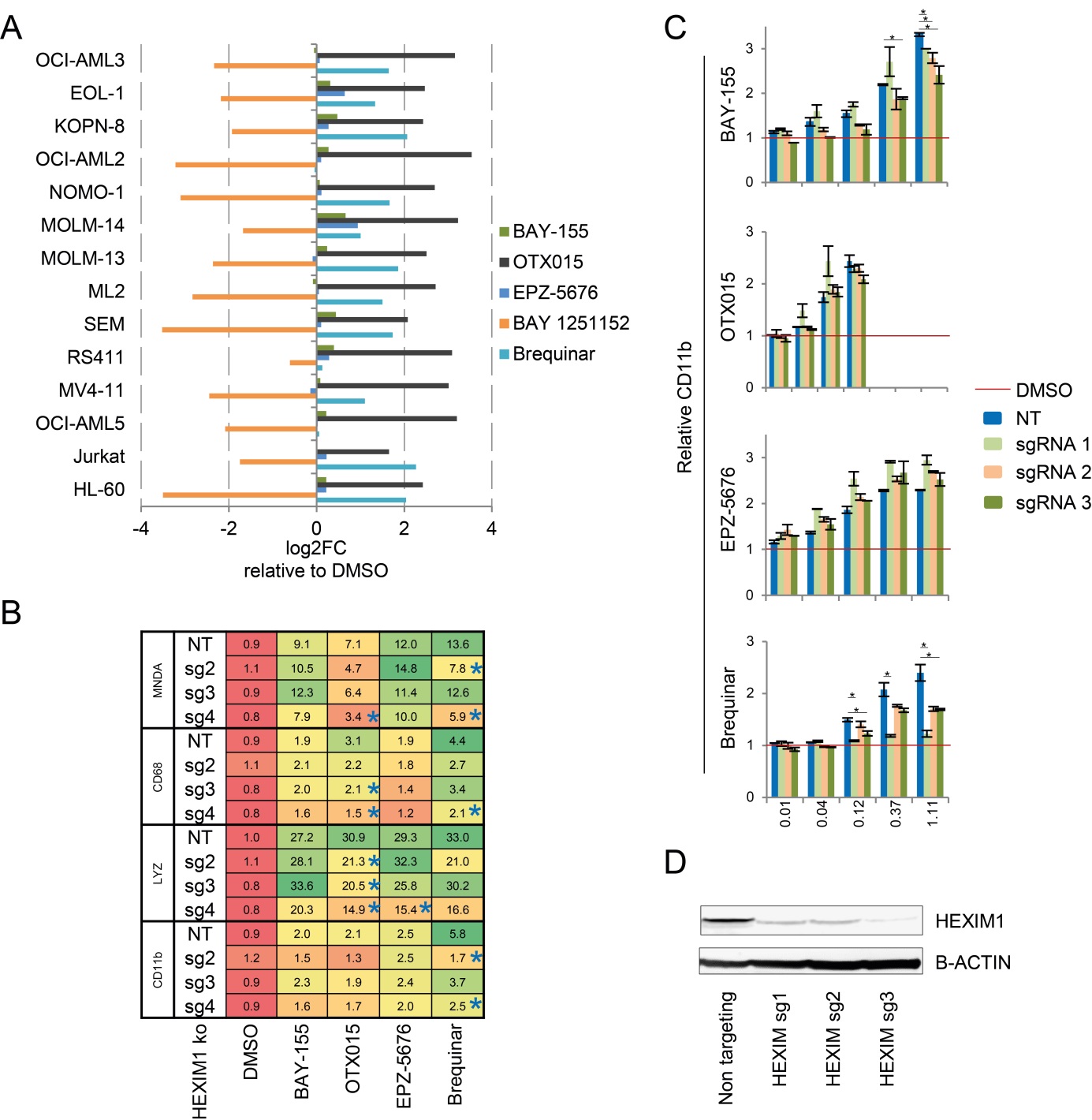


**Figure S6.**

**HEXIM1 links DHODH inhibition with cell differentiation.** (A) Log2FC of *HEXIM1* expression upon inhibitor treatment relative to DMSO. (B) qRT-PCR analysis of representative surface markers and genes associated with hematopoietic differentiation after 7 days of treatment with BAY-155, (3 µM) OTX015 (0.2 µM), EPZ-5676 (5 µM) and Brequinar (3 µM). Analysis was performed in four transgenic lines in MOLM-13 background with HEXIM1 knockout (sg1-3) or a non-targeting sgRNA control (NT). Obtained data is normalized to vehicle (DMSO) and average of three biological replicates is shown. * P<0.05, two sided t-test. (C) Quantification of CD11b expression after titration with BAY-155, OTX015, EPZ-5676, BAY 1251152 and Brequinar in four transgenic MOLM-13 cell lines after 4 days of treatment detected with flow cytometry. Data presented is normalized to vehicle control (DMSO). (D) Western blot analysis of HEXIM1 and β-ACTIN in four MOLM13 transgenic cell lines with HEXIM1 knockout (sg1-3) or a non-targeting control (NT).

**
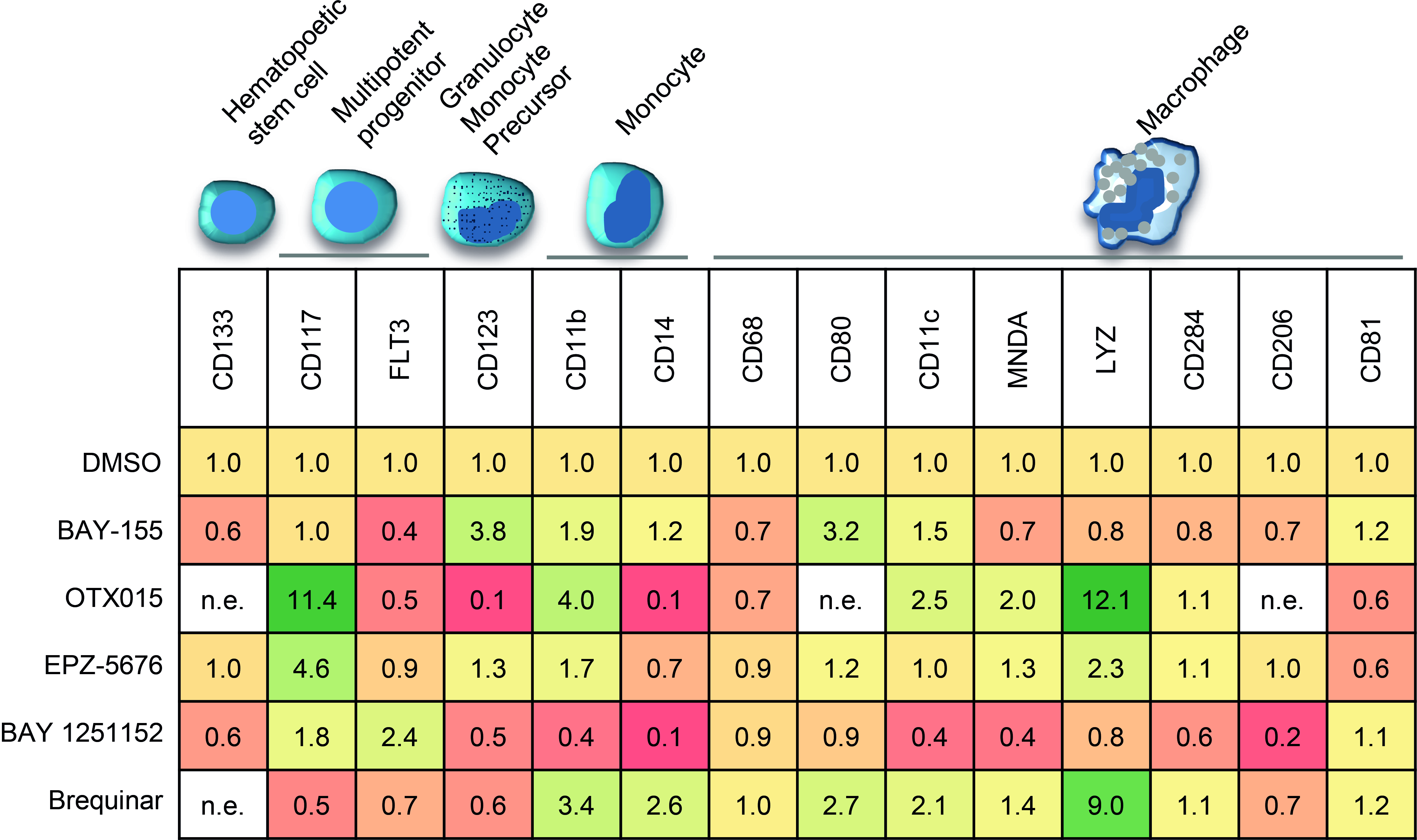
**

**Figure S7.**

**Surface marker analysis after inhibitor induced differentiation in HL-60 cells.** qRT-PCR analysis of representative surface markers and genes associated with hematopoietic differentiation in HL-60 cells after 7 days of treatment with BAY-155, (3 µM) OTX015 (0.2 µM), EPZ-5676 (6 µM), BAY 1251152 (0.1 µM) and Brequinar (1 µM). Data presented is an average of three biological replicates normalized to vehicle control (DMSO). n.e. - not expressed.


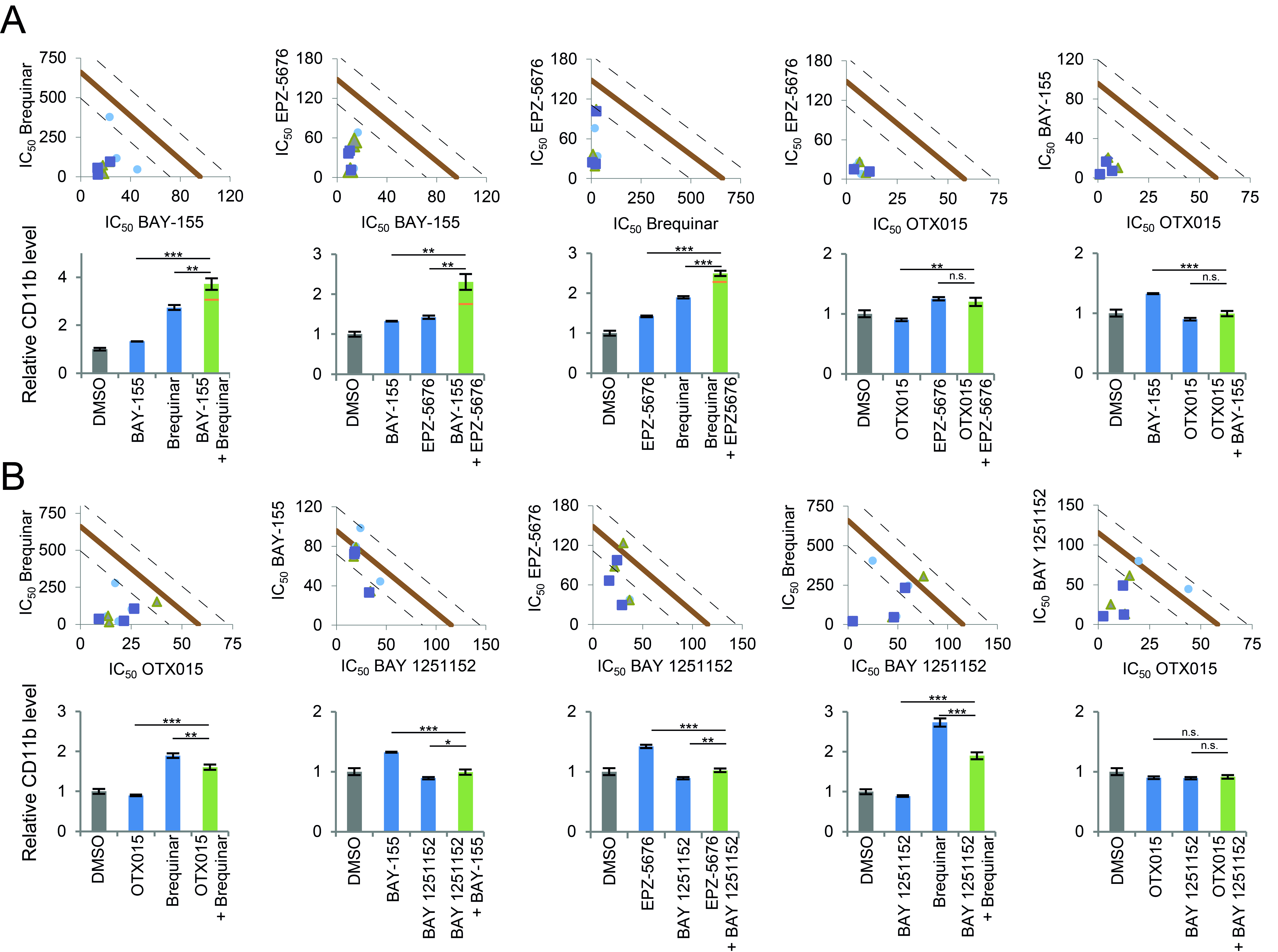


**Figure S8.**

**Analysis of combination effects of used inhibitors in MOLM-13 cells.** IC_50_ - based isobologram analysis (top) and CD11b expression level (bottom) of Brequinar (0.64 µM) - BAY-155. (0.15 µM), EPZ-5676 (0.64 µM) - BAY-155. (0.15 µM), EPZ-5676 (0.64 µM) - Brequinar (0.15 µM), EPZ-5676 (0.15 µM) - OTX015 (0.15 µM), BAY-155. (0.15 µM) - OTX015 (0.15 µM) combinations in MOLM-13 cells after 4 days of treatment. (B) IC_50_ - based isobologram analysis (top) and CD11b expression level (bottom) of Brequinar (0.15 µM) - OTX015 (0.15 µM), BAY-155. (0.15 µM) - BAY 1251152 (0.04 µM), EPZ-5676 (0.64 µM) - BAY 1251152 (0.04 µM), Brequinar (0.64 µM) - BAY 1251152 (0.04 µM), BAY 1251152 (0.04 µM) - OTX015 (0.15 µM) combinations in MOLM-13 cells after 4 days of treatment. The diagonal lines indicate additivity. Experimental data points, represented by dots (square, triangle and circle) indicate biological replicates. Pale red line shows predicted additive effect, dotted lines show additivity area (left). Bar charts (right) show an average of three biological replicates, orange line indicates predicted additive effect, error shows SD, statistics ***P<0.001, **P<0.01, *P<0.05, n.s.P <0.05, two sided ttest.


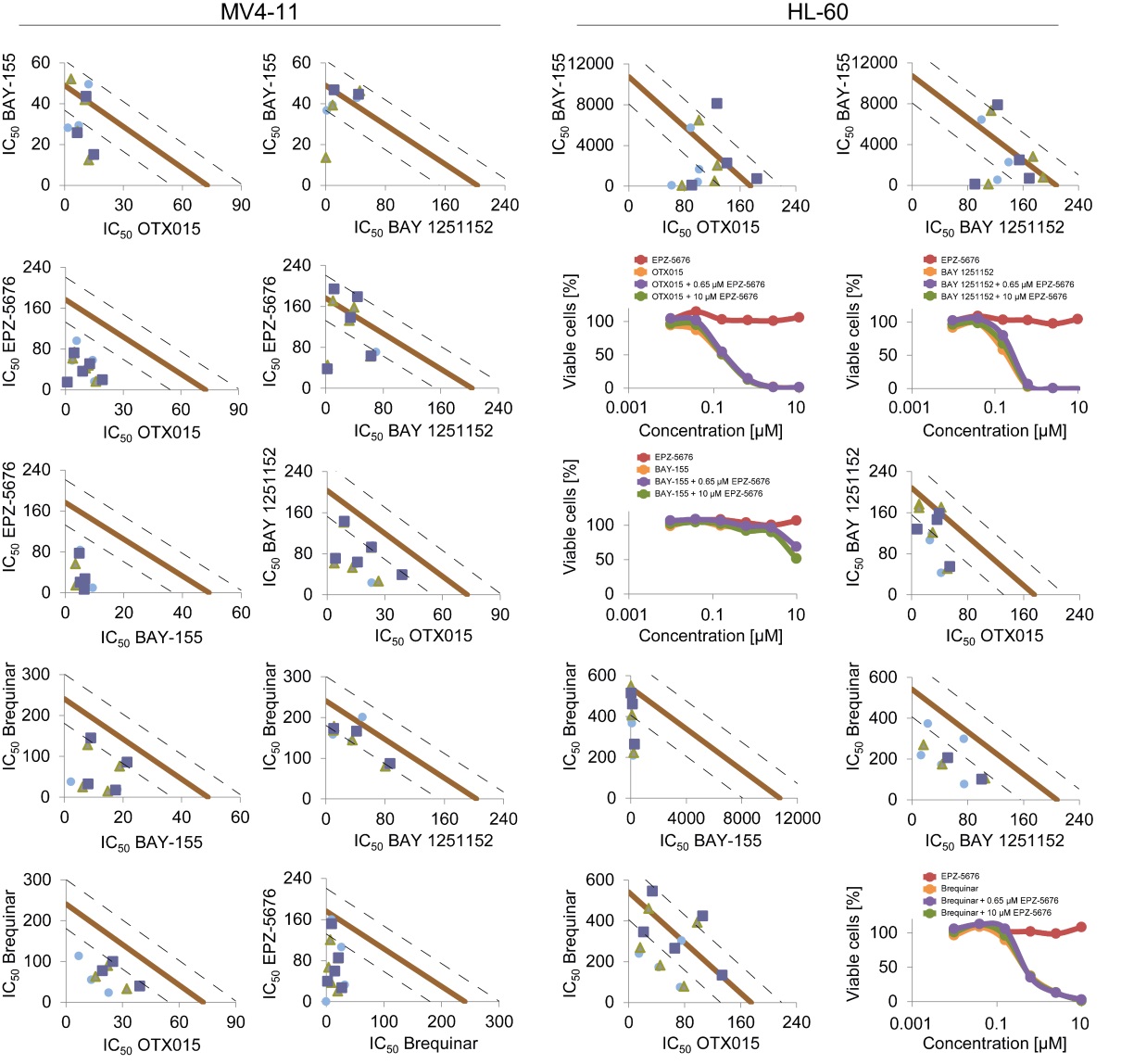


**Figure S9.**

**Analysis of combination effects on proliferation of MV4-11 and HL-60 cells.** Isobologram plots show three biological replicates (indicated by square, triangle and circle), pale red line shows predicted additive effect, dotted lines show additivity area. For inhibitors where IC_50_ determination was beyond concentration range (>10 µM), % of viable cells for single inhibitor treatment and combination was used.
