# Supplementary material for: Functional diversity of inhibitors tackling the differentiation blockage of MLL-rearranged leukemia
Source: J Hematol Oncol. 2019 Jun 28;12:66. doi: 10.1186/s13045-019-0749-y (PMC6599250; doi:10.1186/s13045-019-0749-y)
Supplement: Supplementary file 2 — TaqMan probes used in this study. (DOCX 13 kb) [file 13045_2019_749_MOESM2_ESM.docx]

**Material and Methods**

**TaqMan probes used in this study**

qRT-PCR analysis was performed using the ITGAM (Hs00167304_m1), CD11c (Hs00174217_m1), CD80 (Hs01045161_m1), CD68 (Hs02836816_g1), CD34 (Hs02576480_m1), CD123 (Hs00608141_m1), CD117 (Hs00174029_m1), CD133 (Hs01009259_m1), CD81 (Hs01002167_m1), CD206 (Hs00267207_m1), CD284 (Hs00152939_m1), LYZ (Hs00426232_m1), MNDA (Hs00935905_m1), CD14 (Hs02621496_s1), FLT3 (Hs00174690_m1), 18s RNA (4319413E), hu Cyc (Hs99999904_m1) TaqMan probes (Thermo Fisher Scientific, Waltham, Massachusetts, USA). Obtained data was normalized to 18s RNA or hu Cyc and to the respective DMSO control.
